# Kinematic analysis of activities of daily living performance in frail elderly

**DOI:** 10.1186/s12877-022-02902-1

**Published:** 2022-03-23

**Authors:** Stephanie Schmidle, Philipp Gulde, Sophie Herdegen, Georg-Eike Böhme, Joachim Hermsdörfer

**Affiliations:** 1 Human Movement Science, Department of Sport and Health Sciences, Technical University of Munich, Munich, Germany; 2 Center for Clinical Neuroplasticity Medical Park Loipl, Bischofswiesen, Germany; 3 Lehelmed GmbH General Practitioners Lehel and Medical Center Motorworld, Munich, Germany

**Keywords:** Activities of Daily Living, Frailty, Kinematic Analysis, Wearables, Accelerometry

## Abstract

**Background:**

Frailty is accompanied by limitations of activities of daily living (ADL) and frequently associated with reduced quality of life, institutionalization, and higher health care costs. Despite the importance of ADL performance for the consequence of frailty, movement analyses based on kinematic markers during the performance of complex upper extremity-based manual ADL tasks in frail elderly is still pending.

The main objective of this study was to evaluate if ADL task performance of two different tasks in frail elderlies can be assessed by an activity measurement based on an acceleration sensor integrated into a smartwatch, and further to what degree kinematic parameters would be task independent.

**Methods:**

ADL data was obtained from twenty-seven elderly participants (mean age 81.6 ± 7.0 years) who performed two ADL tasks. Acceleration data of the dominant hand was collected using a smartwatch. Participants were split up in three groups, F (frail, *n* = 6), P (pre-frail, *n* = 13) and R (robust, *n* = 8) according to a frailty screening. A variety of kinematic measures were calculated from the vector product reflecting activity, agility, smoothness, energy, and intensity.

**Results:**

Measures of agility, smoothness, and intensity revealed significant differences between the groups (effect sizes combined over tasks η^2^_p_ = 0.18 – 0.26). Smoothness was particularly affected by frailty in the tea making task, while activity, agility, a different smoothness parameter and two intensity measures were related to frailty in the gardening task. Four of nine parameters revealed good reliability over both tasks (*r* = 0.44 – 0.69). Multiple linear regression for the data combined across tasks showed that only the variability of the magnitude of acceleration peaks (agility) contributed to the prediction of the frailty score (R^2^ = 0.25).

**Conclusion:**

The results demonstrate that ADL task performance can be assessed by smartwatch-based measures and further shows task-independent differences between the three levels of frailty. From the pattern of impaired and preserved performance parameters across the tested tasks, we concluded that in persons with frailty ADL performance was more impaired by physiological deficiencies, i.e., physical power and endurance, than by cognitive functioning or sensorimotor control.

## Introduction

Life expectancy rapidly increases in virtually all developed countries [1], which consequently leads to a larger number of older people as well as an altered ratio between young and old. Old age and its complex processes is known to be accompanied by many geriatric phenomena, like multimorbidity [2] disability [3], and frailty [4], which are, as they are quite unspecific concepts, highly interrelated. The phenomenon of frailty has increasingly received attention during the past decades, as it has been shown to be linked to adverse health outcomes including falls, delirium, institutionalization, and mortality [4–6]. In a broader sense, frailty is understood as a complex concept consisting of various physical, cognitive, nutritional, and social factors [7, 8], representing a high burden for affected individuals, formal and informal caregivers as well as health care systems [9]. According to the well-known standardized phenotype of frailty by Fried et al. [4], the following five criteria are assessed to determine frailty status: unintentional weight loss, exhaustion, slow walking speed, low grip strength, and low physical activity. To be classified as *frail*, at least three criteria must be present. In contrast, the presence of one or two indicators is categorized as *pre-frail*, whereas the absence of any indicator is termed *robust*. It is assumed that frailty among older persons is a dynamic process which is characterized by frequent transitions between frailty states over time. Within this context, transitions to states of advanced frailty are more common than vice versa (hysteresis) [6].

Therefore, early detection of risks associated with the aging process is important to minimize and/or slow down its negative consequences [10]. In case of frailty, this becomes particularly clear when we take a closer look at the disease-related risks that threaten independency of daily living in older people. Elderly people, categorized as frail, show an elevated risk of disability [5, 11, 12]. Thus, frail individuals demonstrate higher rates of developing or worsening disabilities in mobility as well as in basic (b-ADL) and instrumental (i-ADL) activities of daily living over time. These associations can equally be observed among pre-frail elderly, though with a lower magnitude of effect (e.g., [5, 13–16]). ADL typically involve various self-care activities with different degrees of complexity. In general, b-ADL are defined as ‘activities essential for an independent life or necessary for survival, representing everyday tasks required for self-care’ [10]. Whereby i-ADL cover somewhat a more complex set of behaviors [17] and are more sensitive to early cognitive decline [18]. Limitations of b-ADL are frequently measured using the Katz ADL scale [19] or the Barthel Index [20]. I-ADL, on the other hand, are commonly assessed by the Lawton & Brody scale [17]. Changes in ADL performance and especially altered daily activity levels are associated with poor quality of life, increased health care costs, higher mortality, and institutionalization [18]. Furthermore, they can provide important information regarding functional and cognitive abilities, loss of autonomy, and deterioration in health status [21]. Macklai and colleagues [13], as an example, illustrated in their study involving 11,015 community-dwelling men and women, that at 2-year follow up, frail individuals had odds ratios of developing disability in mobility OR 3.07 (95% CI 1.02–9.36), i-ADL OR 5.52 (95% CI 3.76–8.10), and b-ADL OR 5.13 (95% CI 3.52–7.44). Thus, deficits of all types of ADL are associated with frailty and these deficits are of highest relevance.

Sensitive and objective assessment of ADL meets several methodological challenges. This is partly due to the fact that the performance of ADL and especially i-ADL often involves complex sequences of actions and a large amount of degrees of freedom in the strategies and ways to execute those [22]. In manual ADL, such as preparing meal, action sub steps like transporting, grasping, rotating, circling, or balancing, repeatedly alternate with each other and with phases of inactivity [23]. The analysis of ADL performance is in general often limited to subjective scoring of health professionals or the individuum itself, with scores as indicated above. In addition, performance is quantified by measuring the duration of activities, such as walking a certain distance or executing a task. Questionnaire-based methods are subjective, and the time-based assessment does not necessarily represent the characteristics of the movements and the underlying causes for such alterations. It has therefore been concluded that the quantification of movement characteristics employing kinematic analyses may be critical for measuring the extent of frailty [24]. So far, the evaluation of ADL in frail elderly is primarily covered by subjective questionnaires (see above) and approaches involving kinematic analyses of ADL have been mainly focused on lower extremity measures [24], such as various gait parameters and posture, or analyzed kinematics of the upper extremity as secondary outcome. Kubicki and colleagues [25], for example, investigated postural control during self-generated perturbations (rapid focal arm-raising movement towards a target) in frail older adults. Hand kinematics were measured via a Vicon motion capturing system. According to their results, compared to healthy controls, frail participants showed slower hand movements accompanied with delayed postural control, with the latter deficit being the main finding of this study.

Both video analyses and kinematic approaches have been used in studies of upper limb ADL characteristics in various neurological conditions. Investigations of disturbances of ADL in the context of apraxia in stroke or dementia have evaluated videos of execution with respect to defined criteria [26–31]. These studies revealed characteristic error profiles for the different diseases depending on the ADL under examination. However, they are also time-consuming and request expertise to achieve sufficient test reliability. Also, they lack information about speed and fluency of motor execution. In studies utilizing the kinematic approach, trajectories of hand movements were recorded using motion capture methods during execution of a variety of ADL in the context of aging, spinal cord injury as well as in stroke and dementia patients [22, 32–40]. These studies revealed increases in task duration combined with decreases of speed and increased ratios of inactivity as well as more segmented velocity profiles with multiple peaks in stroke patients and elderly compared to young participants. Thus, young participants tended to be faster in task performance than elderly participants and the elderly participants tended to be faster than stroke survivors. Additionally, the movements of young subjects covered less distance compared to the other groups. This suggests that e.g., distance and duration are not only influenced by stroke but also by the aging process. Therefore, kinematic analyses of ADL revealed detailed characteristic performance patterns with high precision and sensitivity.

Recent advances in wearable technologies and data processing technologies have opened new opportunities for the development of practical and automated tools [41] to perform clinical screening in the natural environment outside the laboratory (e.g., [42–45]). Activity tracking systems based on inertial measurement units (IMUs) are small and mobile and enable kinematic analyses independent of a special lab environment. Recently, an approach to assess the dimensions of frailty, as it was characterized by Fried [4], in hand movements in the geriatric population using IMU-based wrist sensors has been introduced [43]. By performing repetitive elbow flexion movements, IMU signals were processed to represent information on the frailty criteria slowness, weakness, and exhaustion. The results showed that there was clear difference in speed of flexion (slowness), power of movement (weakness), and speed variation (exhaustion) between elderly participants associated to the three frailty stages according to the Fried criteria.

Despite the importance of ADL performance for the consequence of frailty (see above), to date and to the best of our knowledge, a movement analysis based on kinematic markers during the performance of complex upper extremity-based manual ADL tasks in frail elderly is still pending. Thus, the main objective of this experimental study was to analyze manual ADL task performance for two different tasks assessed by unilateral activity measurements based on an acceleration sensor integrated into a wrist-worn smartwatch. In a second step, we analyzed whether these measures differ between individual stages of frailty. Additionally, to better understand hand kinematics in the context of frailty, we evaluated possible interaction effects and the effect of task to gain a more comprehensive understanding of the interplay between frailty and task and task-related differences between parameters. Using accelerometry assessment we envisioned a proof-of-concept for this wearable-based approach, which avoids the need for direct observation, video recording, laborious and expensive equipment, and is not bound to laboratory testing. Further, with the outlook of assessments in daily life without standardized tasks, we were interested to examine to what degree kinematic parameters would be task independent. Accordingly, we tested two different ADL in a group of seniors without or with different degrees of frailty. A report which mainly assessed the technical aspects of the approach in a sub-group of the participant has recently been published elsewhere [46].

## Materials and Methods

### Participants

**Fig. 1:**
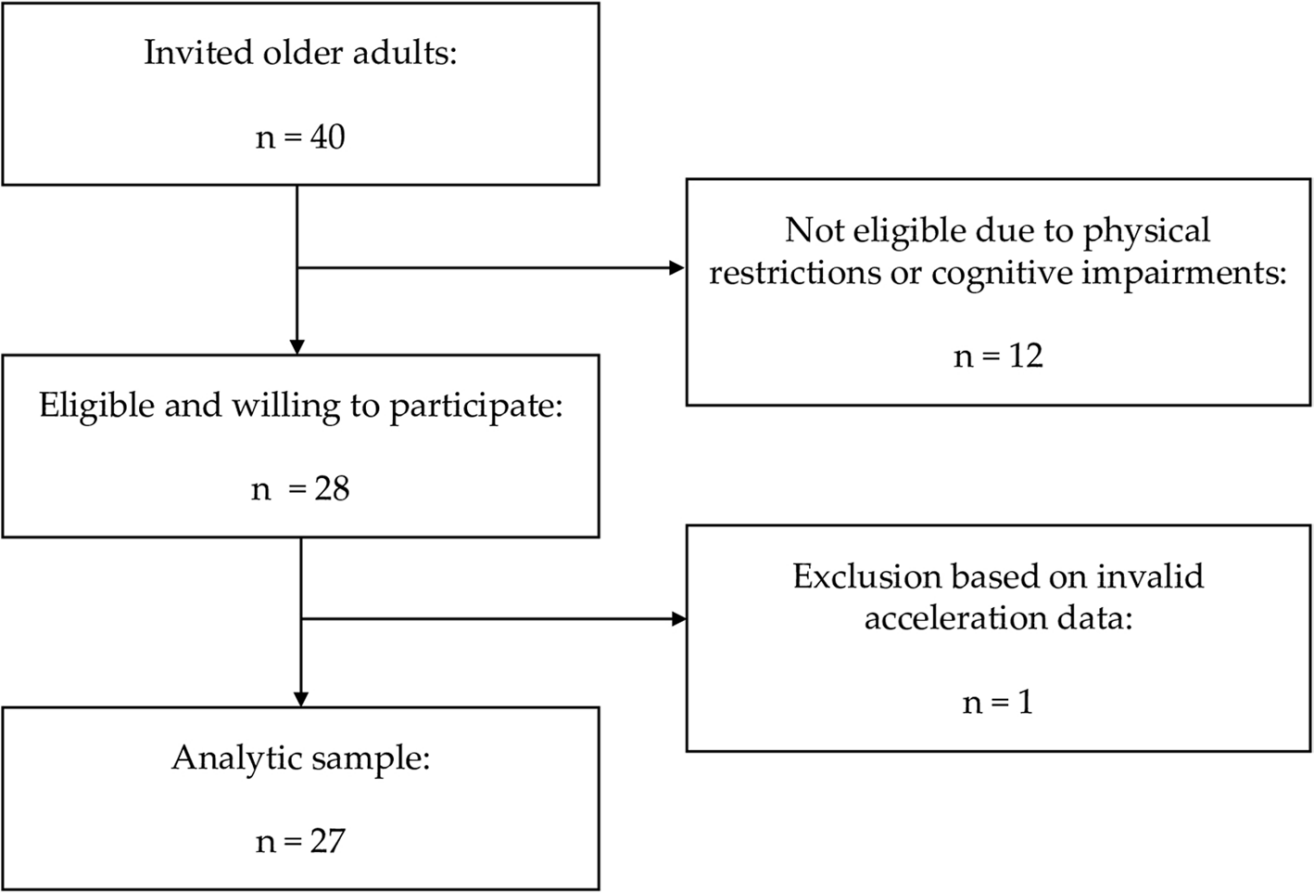
Flowchart of the recruitment procedure

Twenty-seven older adults aged 60 years and older (min – max: 68 – 96 years) participated in this study. Subjects were recruited from care institutions and the community. Inclusion criteria for participation were defined as a minimum age of 60 years and a score of at least 24 points on the Mini Mental State Examination (MMSE) [47]. Elderly people with cognitive impairments (< 24 MMSE) or severe neurological conditions were excluded (see Fig. 1). Ethical approval was given by the ethics committee of the Medical Faculty of Technical University of Munich. All participants gave written informed consent.

A posthoc power analysis (G*Power 2) for ‘MANOVA: Repeated measures, within-between interaction resulted in a power of > 0.99 [48], indicating a sufficient sample size for our approach.

To assess the frailty status of each participant, an adapted version of the Fried frailty score was applied according to Kunadian et al. [49]. Similar to the original Fried score (see Introduction), the performance on five criteria ‘weight loss’, ‘exhaustion’, ‘low physical activity’, ‘low grip strength’, and ‘slow walking speed’ was assessed. With failures in one or two criteria, a person was categorized as ‘pre-frail’ (P), above as ‘frail’ (F), and with no failure as ‘robust’ (R). Table 1 shows the resulting number of participants in each frailty category as well as the participants’ age, anthropometrics, MMSE, grip strength, timed up & go test (TUG), and sex. Eight participants had no positive criterion and were thus in the group of robust. From the 13 participants of the pre-frail group, six scored ‘1’ and seven scored ‘2’. From the six participants of the frail group, three scored ‘3’ and three scored ‘4’ Fried points. Statistical analysis for differences between the subgroups revealed no difference for sex, height, weight, BMI, and MMSE but a statistical difference for age, grip strength, and the TUG (one-way ANOVA: age, height, weight, BMI, MMSE, grip strength, and TUG; and Pearson Chi-Squared test: sex). Pairwise post hoc tests (Tukey tests) confirmed age differences between the frail group and the two other groups (R-F *p* = 0.003, P-F *p* = 0.004), but no significant difference between the age of the robust and the pre-frail group. For grip strength, there was a significant difference between the robust and the frail group (R-F *p* = 0.009) and a tendency between pre-frail and frail participants (P-F *p* = 0.081). Furthermore, the TUG differed between the robust and the frail group (R-F p = 0.025). Three of the six individuals classified as frail were unable to perform the TUG test at all.

**Table 1:** Descriptive statistics of the subsamples

Characteristics	Robust R	Pre-frail P	Frail F	Total	P-value	Effect Size
	*n* = 8	*n* = 13	*n* = 6	*n* = 27		
**Female, n (%)**	6 (75)	7 (54)	5 (83)	18 (67)	0.304	V 0.291
**Age (years)**	78.4 (6.5)	79.8 (5.6)	89.8 (4.2)	81.6 (7.0)	0.002*	n^2^_p_ 0.413
**Height (cm)**	165.0 (6.6)	167.4 (9.1)	164.3 (12.3)	166.0 (9.0)	0.750	n^2^_p_ 0.024
**Weight (kg)**	76.1 (23.6)	80.8 (15.9)	83.8 (16.3)	80.1 (18.0)	0.728	n^2^_p_ 0.026
**BMI (kg/m**^**2**^**)**	27.9 (8.4)	28.6 (4.0)	31.3 (7.8)	29.0 (6.3)	0.586	n^2^_p_ 0.044
**MMSE**	28.1 (1.9)	27.5 (2.0)	25.8 (1.9)	27.3 (2.1)	0.111	n^2^_p_ 0.167
**Grip strength (kg)**	24.4 (7.2)	19.4 (8.3)	10.8 (7.2)	19.0 (9.0)	0.012*	n^2^_p_ 0.308
**TUG (sec)**	11.9 (4.4)	20.7 (11.5)	30.3 (9.5)	18.9 (10.9)	0.023*	n^2^_p_ 0.302

Mean values, standard deviations, and p-values of sample comparison (an asterisk indicates a statistically significant group effect). Effect Sizes: η^2^_p_ partial eta squared and Cramer’s V. *BMI* Body mass index, *MMSE* Mini Mental State Examination, *TUG* timed up & go test

### Task and procedure

**Fig. 2:**
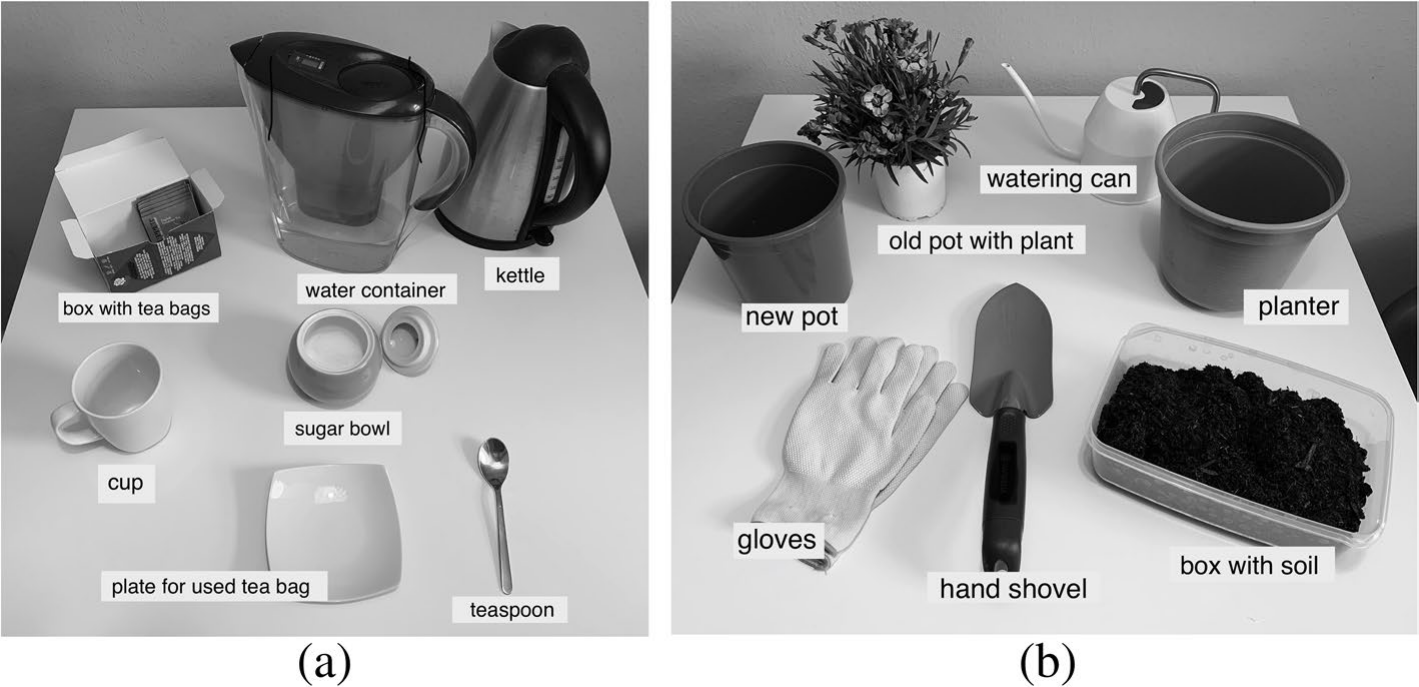
(**a**) The setting of the TEA task with the associated tools; (**b**) The setting of the GARDEN task with the associated tools

The measurements were conducted in the participants’ homes or in the respective institutions. Each subject received verbal explanation of the procedure in advance. After completion of the demographics form, MMSE and frailty status were assessed. ADL performance was measured in standing position (if possible) behind a table with all items placed in front of every participant in a standardized way (Fig. 2). Participants who were not able to stand were allowed to sit on a chair. Post hoc inspection of their results did not reveal any obvious discrepancy with the standing participants.

#### Activities of daily living

Participants were instructed to perform two different ADL. Each ADL was performed once in a pseudorandomized order. The ADL tasks were to prepare a cup of tea (tea making – TEA) or to replant a plant (gardening – GARDEN), see Fig. 2.

In TEA, the following items were given: water container with approximately 250 ml of room-temperatured water, a kettle, a paper box filled with tea bags, a bowl of sugar, a plate to remove and place used tea bags, a cup, and a teaspoon. Standardized instruction was given to each participant as follows:

‘*Can you prepare a cup of tea with one spoon of sugar, standing behind the table? Please execute the task in a natural way, as you would do it at home and in a speed and a way which is appropriate for you’.*

In GARDEN, the following items were given: a box of soil, a water can filled with approximately 500 ml of water, a plant, a pot, a planter, gloves, and a hand shovel. Standardized instruction was given to each participant as follows:

*‘Can you replant this plant into the pot and water it, standing behind the table? Please execute the task in a natural way, as you would do it at home in a speed and a way which is appropriate for you’.*

During the ADL performance, the hand movement of the dominant hand was captured using a Huawei 2 (4G) smartwatch attached with a size-adjustable velcro strap.

#### Sensor-based kinematic parameters

Sampling frequency of the 3-dimensional acceleration signals was 100 Hz. The absolute acceleration vector was calculated by the Euclidean mean. The gravitation was subtracted as a constant (see Discussion), and the signal was smoothed using a 420 ms local regression algorithm [50]. The analysis was based on kinematic parameters using acceleration data identified in our previous study [46]. Two additional parameters (*Acceleration per Second (APS)* and *95*^*th*^* Percentile of Acceleration Peaks (MAX95))* were included to have supplementary parameters for energy expenditure and an outlier-robust measure of speed/intensity. Additionally, parameters were grouped according to their intention of measure (activity, agility, smoothness, energy, and intensity). All data processing was performed using MatLab R2020a (The MathWorks Inc., Natick, MA, USA).

#### Activity

- *Trial Duration (TD):* Time to execute the task in seconds. The start and end was triggered by the investigator by starting and stopping the sensor recording.
- *Relative Activity (RA):* Period of time in which the absolute acceleration signal exceeded 0.2 m/s^2^ related to TD. It ranges from > 0.0 to 1.0, with 1.0 indicating the absence of any phases of inactivity.

#### Agility

- *Peak Standard Deviation (STD):* Standard deviation of all acceleration peaks (local maxima) in m/s^2^. This parameter intends to reflect the intensity of actions with high values reflecting more agile movement execution. Low values represent a rather peculiar monotone behavior.

#### Smoothness

- *Peaks Per Second (PPS):* Number of acceleration peaks per second as a measure of movement smoothness.
- *Peak Ratio (RATIO):* Ratio between the number of acceleration peaks with a minimum prominence of 0.2 m/s^2^ and the total number of acceleration peaks. A measure of movement smoothness reflecting the amount of distinct movements relative to all movements including noise.

#### Energy

- *Weighted Sum of Acceleration per Second (SUM):* Temporal mean of squared acceleration. A parameter to (non-linearly) estimate energy expenditure. Noise and small movements were intended to be weighted less by using the square of acceleration.
- *Acceleration per Second (APS):* Absolute acceleration per second in m/s^3^ as a measure of energy expenditure.

#### Intensity

- *Mean Peak Acceleration (MPA):* Mean of acceleration peaks as a measure of the intensity of actions adapted from a similar measure of velocity to assess general movement speed/intensity.
- *95*^*th*^* Percentile of Acceleration Peaks (MAX95):* The 95^th^ percentile of all acceleration peaks thought as an outlier-robust measure of movement speed/intensity.

### Statistical Approach

In a first step, a two-way mixed MANCOVA was run to test for an interaction effect between task and frailty status with age as covariate. One-way ANOVAs (Tukey post hoc) were run to compare the above-mentioned kinematic parameters for group (R, P, and F) and task (task average, TEA, GARDEN). In a second step, kinematic parameters were correlated between the two ADL (e.g., RA for TEA and RA for GARDEN) to estimate the task specificity of the measures. Third, it was investigated whether the 5-point-frailty score can be predicted from the performance data. To that aim, kinematic parameters (TEA, GARDEN, and average) were used to model the adapted Fried score by models of multiple linear regression (MLR). For MANCOVA analyses, the R package “MANOVA.RM” including MATS statistics for multivariate data was used as it proved to be independent of distribution of the data and unequal dispersion of covariates between groups [51]. Furthermore, a bootstrap resampling approach (100 k) was used as proposed by Konietschke et al. [52] or Friedrich et al. [53] in case of small sample sizes. Effect-sizes were given in partial eta squared η^2^_p_ and Cohen’s *d*, the critical variance inflation was 5.0, and $\mathrm{\alpha }$ was set to 0.05. All tests were run in SPSS version 26 software (IMB, NY, United States) and R Studio (version 3.5.1, RStudio Inc., Vienna, Austria).

## Results

**Fig. 3:**
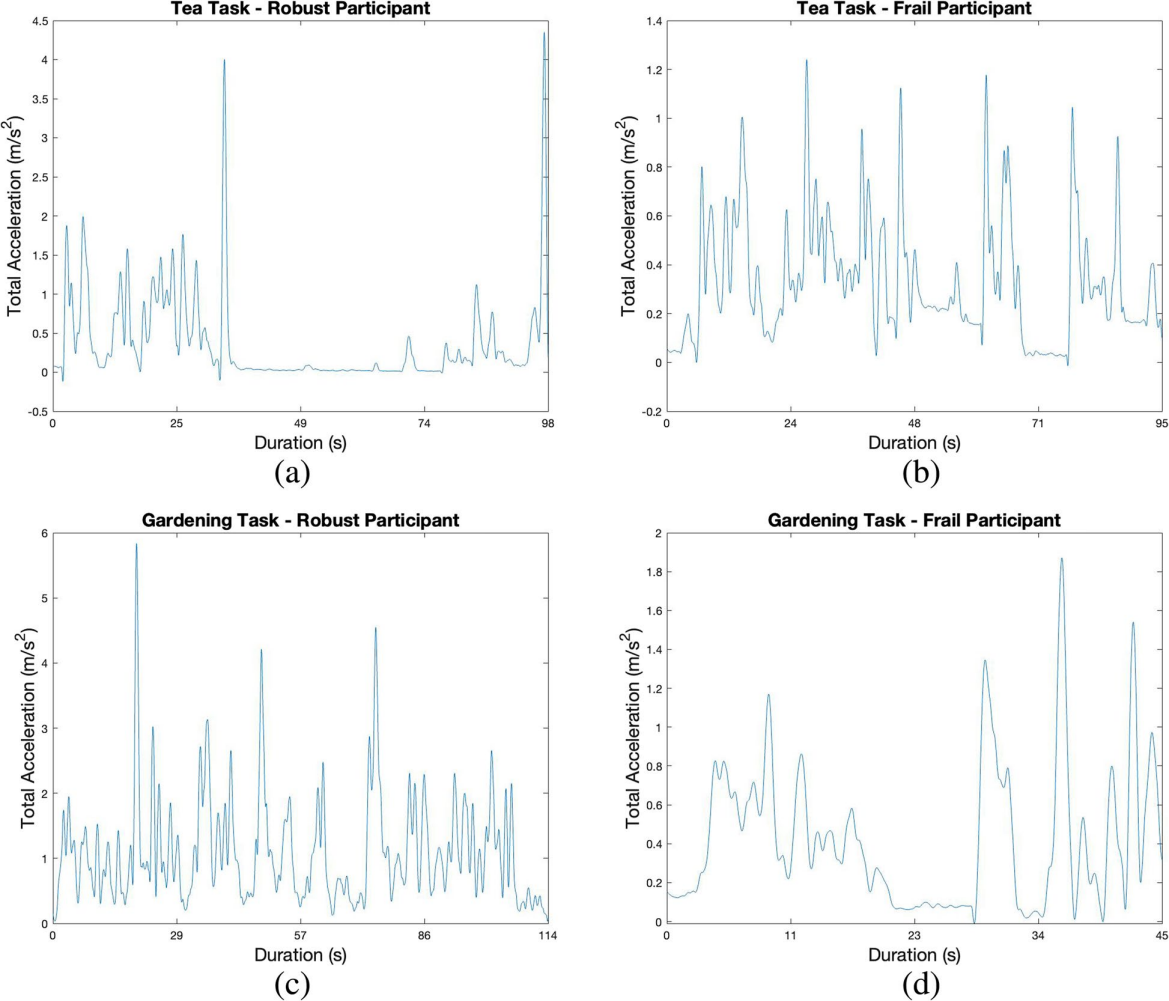
(**a**) Acceleration profile of TEA of a robust elderly woman at the age of 90 years (P26); (**b**) Acceleration profile of TEA of a frail elderly woman at the age of 93 years (P24); (**c**) Profile of GARDEN of participant P26; (**d**) Profile of GARDEN of participant P24

The following plots illustrate the rectified and smoothed (2 s ‘loess’ filtered absolute accelerations for illustration purposes) acceleration profiles of a frail and non-frail participant for the GARDEN and the TEA task. Profile a) (Fig. 3) shows the movement graph with the majority absolute accelerations between 0.5–1.0 m/s^2^ and maximal peaks of about 4 to 4.5 m/s^2^. The profile illustrates a pause between second 40 and 70 most probably indicating a segment of the boiling phase of the water (expected standardized boiling time of 60 s for each participant). The activity graph of the frail participant (Fig. 3b) shows total accelerations primarily between 0.2 and 0.6 m/s^2^. The acceleration peaks achieved values of up to 1.2 m/s^2^. It is noticeable, that there seems to be no clear movement pause during the boiling period of the water. The total duration between the frail and non-frail person in this case was quite equal. Profiles c) and d) illustrate the GARDEN task. Again, there are obvious differences in the magnitude of acceleration between the non-frail and frail participant, with values as high as 6 m/s^2^ compared to approximately 2 m/s^2^. Additionally, the frail person executed the task in less time (45 s vs. 114 s).

Many (but not all) of these findings turned out to be representative for the groups of participants, as shown below.

### Group differences and Inter-task correlations

**Fig. 4:**
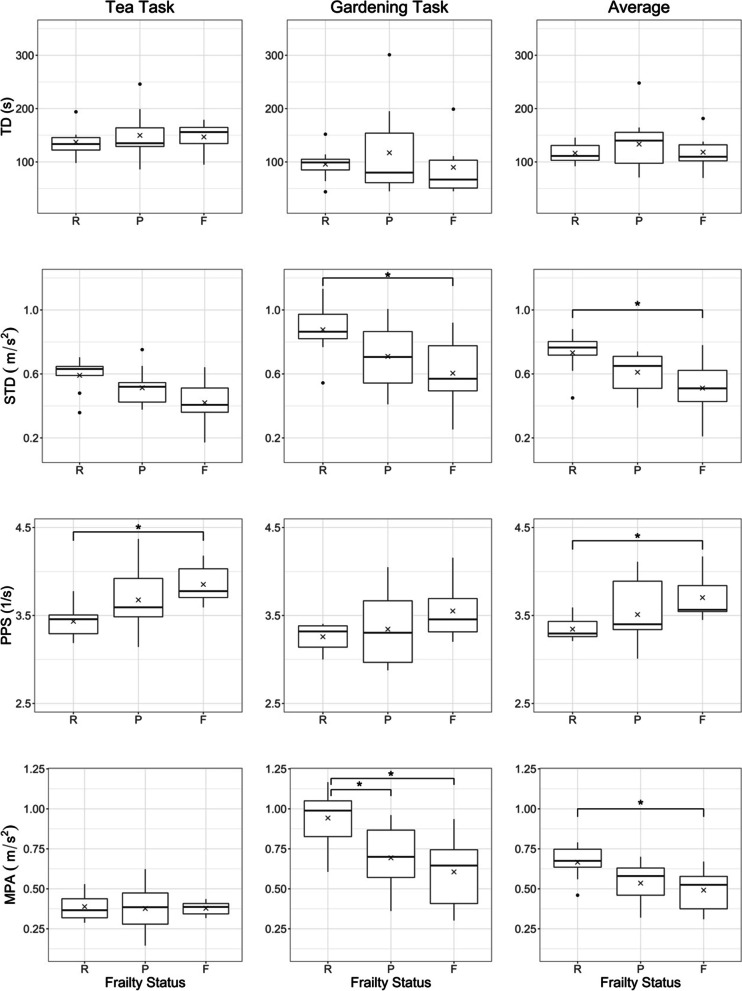
Boxplots of four kinematic parameters for TEA, GARDEN, and task average for the frailty status robust (R), pre-frail (P), and frail (F). *TD* Trial Duration as a measure of activity, *STD* Peak Standard Deviation (‘agility’), *PPS* Peaks Per Second (‘smoothness’), *MPA* Weighted Sum of Acceleration per Second (‘intensity’), * statistically significant group effect (< 0.05), *x* group means, *error bars* standard error. For all measures besides TD, significant group effects were found

Our initial MANCOVA revealed no significant impact of age. Therefore, we reanalyzed the data using a MANOVA. The analysis showed significant main effects of frailty status (group) and task and no significant interaction effect of frailty status and task (see Table 2). Table 3 shows the main effect of group and task for each kinematic parameter. Table 4 illustrates the results of the post hoc ANOVAs for the group effect for the two tasks separately. Figure 4 provides graphical representations of the results for four selected parameters.

**Table 2:** Results of MANOVA

Effect	F (18.000, 34.000)	p-Value
Group	55.856	0.03*
Task	320.446	< 0.01*
Group x Task	36.078	0.12

^*^Significant effect is reached

**Table 3:** Main effects of group (robust, pre-frail, frail) and task (TEA, GARDEN) for the kinematic parameters

Parameter		Activity		Agility	Smoothness		Energy		Intensity	
		TD (s)	RA (-)	STD (m/s^2^)	PPS (1/s)	RATIO (-)	SUM (m^2^/s^5^)	APS (m/s^3^)	MPA (m/s^2^)	MAX95 (m/s^2^)
Group	R	116 (21)	0.57 (0.04)	0.73 (0.14)	3.3 (0.1)	0.55 (0.05)	23.87 (6.33)	21.05 (3.12)	0.67 (0.11)	1.39 (0.26)
	P	134 (46)	0.55 (0.10)	0.61 (0.12)	3.5 (0.3)	0.49 (0.08)	25.20 (13.90)	19.94 (5.20)	0.54 (0.12)	1.18 (0.24)
	F	118 (38)	0.56 (0.08)	0.51 (0.20)	3.7 (0.3)	0.47 (0.09)	26.39 (11.17)	21.74 (4.00)	0.50 (0.14)	1.01 (0.37)
	p	0.56	0.92	0.03*	0.04*	0.08	0.92	0.69	0.03*	0.06
	η^2^_p_			0.26	0.18				0.26	
Task	Tea	145 (35)	0.44 (0.13)	0.52 (0.14)	3.6 (0.3)	0.34 (0.10)	25.23 (10.44)	17.88 (6.17)	0.38 (0.11)	0.93 (0.24)
	Garden	105 (61)	0.67 (0.11)	0.74 (0.22)	3.4 (0.3)	0.67 (0.12)	24.91 (15.63)	23.46 (5.44)	0.75 (0.24)	1.47 (0.46)
	p	0.005*	< 0.001*	< 0.001*	0.003*	< 0.001*	0.93	< 0.001*	< 0.001*	< 0.001*
	η^2^_p_	0.15	0.47	0.28	0.15	0.72		0.19	0.51	0.37

Mean values, standard deviation, *R* robust: ‘0’, P pre-frail: ‘1–2’, F frail: ‘3–5’, p-value, η^2^_p_ partial eta squared. *TD* Trial Duration, *RA* Relative Activity, *STD* Peak Standard Deviation, *PPS* Peaks Per Second, *RATIO* Peak Ratio, *SUM* Weighted Sum of Acceleration per Second, *APS* Acceleration per Second, *MPA* Mean Peak Acceleration, *MAX95* 95^th^ Percentile of Acceleration Peaks

**Table 4:** Kinematic assessment of the tea and gardening task in R (robust: ‘0’), P (pre-frail: ‘1–2’) and F (frail: ‘3–5’)

Parameter		Activity		Agility	Smoothness		Energy		Intensity	
		TD (s)	RA (-)	STD (m/s^2^)	PPS (1/s)	RATIO (-)	SUM (m^2^/s^5^)	APS (m/s^3^)	MPA (m/s^2^)	MAX95 (m/s^2^)
TEA	R	137 (28)	0.40 (0.08)	0.60 (0.11)	3.4 (0.2)	0.36 (0.10)	22.81 (7.76)	16.30 (3.06)	0.39 (0.09)	0.99 (0.19)
	P	150 (42)	0.44 (0.16)	0.51 (0.11)	3.7 (0.4)	0.32 (0.09)	24.15 (11.90)	17.61 (8.27)	0.38 (0.14)	0.95 (0.27)
	F	147 (30)	0.51 (0.12)	0.42 (0.16)	3.9 (0.2)	0.34 (0.05)	30.79 (9.67)	20.57 (2.82)	0.38 (0.05)	0.83 (0.24)
	p	0.74	0.28	0.06	0.04*	0.72	0.33	0.06	0.97	0.47
	η^2^_p_				0.24					
GARDEN	R	96 (32)	0.74 (0.03)	0.88 (0.18)	3.3 (0.2)	0.75 (0.05)	24.93 (9.24)	25.80 (4.43)	0.94 (0.18)	1.78 (0.39)
	P	117 (76)	0.66 (0.11)	0.71 (0.20)	3.3 (0.4)	0.64 (0.12)	26.24 (18.77)	22.26 (5.14)	0.69 (0.20)	1.42 (0.39)
	F	90 (59)	0.61 (0.15)	0.61 (0.24)	3.6 (0.4)	0.60 (0.15)	21.99 (17.05)	22.92 (7.09)	0.61 (0.25)	1.19 (0.50)
	p	0.64	0.03*	0.05*	0.28	0.02*	0.87	0.35	0.01*	0.04*
	η^2^_p_		0.20	0.22		0.23			0.31	0.24
Inter-task correlation	*r*	0.19	-0.15	0.61	0.69	0.16	0.45	0.11	0.11	0.44
	*p*	0.34	0.46	** < .001**	** < .001**	0.54	**0.02**	0.59	0.60	**0.02**

Mean values, standard deviation, p-value, r inter-task correlation, η^2^_p_ partial eta squared. *TD* Trial Duration, *RA* Relative Activity, *STD* Peak Standard Deviation, *PPS* Peaks Per Second, *RATIO* Peak Ratio, *SUM* Weighted Sum of Acceleration per Second, *APS* Acceleration per Second, *MPA* Mean Peak Acceleration, *MAX95* 95^th^ Percentile of Acceleration Peaks

The main effect of task revealed significant differences for the kinematic parameters, F (9.000, 16.000) = 320.446, *p* =  < 0.001. All parameters, except the ‘energy’ measure SUM, showed significant differences between the tasks with effect sizes ranging from 0.15 to 0.72 (η^2^_p_ partial eta squared), see Table 3. Additionally, there was a significant main effect of group, F (18.000, 34.000) = 55.856, *p* = 0.03. Further comparisons showed that the ‘agility’ measure STD, the ‘smoothness’ measure PPS, and the ‘intensity’ measure MPA statistically differed between subjects with frailty scores of ‘0’ (R), of ‘1–2’ (P), and ≥ ‘3’ (F) (see Table 3). Post hoc tests revealed significant differences between robust subjects and subjects who were pre-frail or frail. No significant post hoc test was found comparing the group of pre-frail and frail. The calculated effect sizes for the post hoc comparison tests ranged between -1.73 – 1.40 (Cohen’s d). Smoothness RATIO and intensity MAX95 revealed trends for statistically significant differences between groups (*p* = 0.08 and 0.06), Table 3.

For the TEA task, the parameter PPS, characterizing movement smoothness, significantly differed between the groups, F (2,24) = 3.694, *p* = 0.04, see Table 4 and Fig. 4. A Tukey post hoc test confirmed PPS differences between the frail group and the robust group (F-R p_tukey_ = 0.04), but no significant differences between the PPS values of the robust and the pre-frail group as well as the pre-frail group and frail group. ANOVAs for the STD (‘agility’) and APS (‘movement energy’) revealed trends for differences between groups (*p* = 0.06, see Table 4).

For the GARDEN task, RA (‘activity’), STD (‘agility’), the RATIO (‘smoothness’), and the two measures for movement ‘intensity’ MPA and MAX95 significantly differed between the groups with effect sizes ranging from η^2^_p_ = 0.20 – 0.31 (see Table 4). The RA values for the GARDEN task showed a decreasing tendency between the robust group towards the frailer participants. Post hoc comparison revealed that RA and RATIO only showed differences between the robust group and the pre-frail group (R-P p_tukey_ = 0.05 and 0.03, *d* = 0.91 and 1.07). MPA, on the other hand, showed differences between the robust and the pre-frail group as well as between the robust and the frail group (R-P, R-F Cohen’s *d* = 1.30 and 1.58). STD and MAX95 differed between robust and frail (R-F Cohen’s *d* = 1.30 and 1.33), but not between the pre-frail and the frail group.

Compared to the TEA task, task average trial duration (TD) of the GARDEN task was shorter and the ratio of inactivity (1.0 - RA) was also lower (see Table 4) indicating fewer and shorter breaks compared to the TEA task where the boiling of water frequently resulted in movement breaks (see Figure 4). The measures for ‘energy’ and ‘intensity’ were all clearly higher than in the TEA task (see Table 4). Of the ‘smoothness’ measures, PPS was about equal in both tasks, while RATIO was higher in the GARDEN task.

As could be expected from the significant group effects for the averaged measures, the parameters STD, PPS, SUM, and MAX95 showed significant coefficients of correlation between the TEA and GARDEN task ranging from *r* = 0.44 – 0.69, being particularly high for agility (STD) and smoothness (PPS), see Table 4. In the following figure (Figure 4), TD, STD, PPS and MPA are plotted for each task divided by the three frailty groups.

### Models of multiple linear regression

Multiple linear regressions were performed to predict the frailty score with its maximum 5 levels from the kinematic measures obtained from the execution of the two daily living tasks. The kinematic parameters were added to the regression. The prediction model of the frailty status by the task average explained 25% of variance by the parameter STD, F (2, 24) = 9.593, *p* = 0.005 (see Table 5). The beta weight was -0.527.

**Table 5:** Model of frailty score by parameters of the task average

Parameter	R^2^	p-value	ß-weight	VIF
**Model – Frailty Score**	0.248	0.005		
STD		0.005	-0.527	1.000

*STD* Peak Standard Deviation (m/s^2^), frailty score (0–5)

For the TEA task, the smoothness measure PPS and the activity measure RA statistically added to the prediciton of the frailty model, F (2, 24) = 7.116, p 0.004, explaining 32% (R^2^_adj_) of variance (see Table 6). The beta weights were between 0.332 and 0.512.

**Table 6:** Model of frailty score by parameters of the tea task

Parameter	R^2^ (adjusted)	p-value	ß-weight	VIF
**Model – Frailty Score**	0.320	0.004		
PPS		0.004	0.512	1.000
RA		0.051	0.332	1.000

*PPS* Peaks Per Second (1/s), *RA* Relative Activity (-), frailty score (0–5)

For the GARDEN task, STD statistically added to the prediction of the frailty status, F (2, 24) = 6.855, *p* = 0.015, R^2^ = 0.184. This variable added statistically to the prediction with a beta weight of -0.464 (see Table 7).

**Table 7:** Model of frailty score by parameters of the gardening task

Parameter	R^2^	p-value	ß-weight	VIF
**Model – Frailty Score**	0.184	0.015		
STD		0.015	-0.464	1.000

*STD* Peak Standard Deviation (m/s^2^), frailty score (0–5)

## Discussion

The aim of this experimental study was to investigate complex manual ADL task performance employing kinematic analyses based on unilateral acceleration sensor data from a wrist-worn smartwatch. Furthermore, we wanted to analyze whether these measures differ between individual stages of frailty and to examine to what degree kinematic parameters would be task independent. The findings of the present analysis indicate that task performance can be assessed via acceleration sensors and do show differences in kinematic parameters for the separate levels of frailty.

Our analyses revealed that there was no significant impact of age and no interaction between the ADL tasks (GARDEN and TEA) and the level of frailty of the participants, which was defined by an adopted version of the Fried score [49]. However, there were significant effects of task and group. All parameters except the ‘energy’ measure SUM differed significantly between the tasks with large effect sizes between 0.20 – 0.31 (partial eta squared). The main effect of group revealed differences for ‘agility’ (STD), ‘smoothness’ (PPS), and ‘intensity’ (MPA) measures with lower ‘agility’ (lower STD), less smoothness (higher PPS), and lower ‘intensity’ (MPA) for participants categorized as pre-frail or frail compared to robust participants. Furthermore, four out of nine parameters (STD, PPS, SUM, and MAX95) showed good reliability over both tasks (coefficient of correlation > 0.43). Models of multiple linear regression were able to predict between 0.18 and 0.32 of the variances in the frailty scores of the elderly, depending on the used task kinematics (TEA, GARDEN, combined tasks). For GARDEN and the combined task, STD significantly contributed to the prediction (beta = -0.46 and -0.53). While in TEA, PPS and RA contributed the most to the model (beta = 0.51 and 0.33, p values = 0.004 and 0.051).

There was a clear difference between the tasks. Except for the energy measure SUM (weighted sum of acceleration per second), all parameters showed significant differences between TEA and GARDEN. This might be due to differences in task complexity and requirements [54]. While TEA can be seen as a task requesting precise movements, for example to put the tea bag into the mug, the garden task might demand more energetic and powerful movements with manipulation of heavier objects like the hand shovel or the box with soil. Further group analyses split up for each task separately demonstrated, that for TEA, the smoothness parameter PPS increased with increasing frailty level. MLR revealed that RA and PPS contributed to the prediction. Since smoothness is typically related to movement coordination [23, 55], this finding underpins the assumption, that this task is more dependent on fine motor control. For the GARDEN task, on the other hand, the activity measure RA, the agility measure STD, the smoothness measure PPS, and both intensity measures MPA and MAX95 differed between the groups. Consequently, people with higher level of frailty had more movement pauses during the task execution of GARDEN and less intense and agile movements than the robust ones. This might be related to prolonged planning phases or resting/energy saving under equal duration. MLR showed that only STD (R^2^ = 18%) was a significant factor in the model. Additional analysis for the aspect of strength in GARDEN showed that there was a significant correlation between grip strength and STD (*r* = 0.625, *p* < 0.001). Therefore, GARDEN might be more depending on gross motor control.

Interestingly, trial durations (TD) in both complex ADL were not correlated and showed no differences between subjects with higher (frail, F), intermediate (pre-frail, P), and lower frailty scores (robust, R). In TEA, the frail group needed on average 147 s to complete the tasks, whereas the pre-frail group took 150 and the robust group 137 s to complete. For GARDEN, the average duration for the frail participants was 90 s and for the pre-frail and robust individuals 117 and 96 s. This is particularly striking in relation to previous literature that reported clear increases of the duration of similar ADL tasks in the context of neurological conditions, such as spinal cord injury, stroke, and dementia, but also in regards to aging [22, 32–40]. With reference to this rational, we would have expected to find increased TDs in this cohort as well. However, TD seems to be not a good estimate of general task performance (at a natural pace) for people with different levels of frailty. This might be the case in particular if the accuracy is not controlled [56]. Furthermore, given our experience with increases in TD associated with aging [35, 57] and neurological diseases [22] in similar tasks, we hypothesized that these prolongations are due to cognitive aspects of the tasks. In a previous study of our work group [57], young and healthy older adults had to execute a quite similar tea task. The elderly participants committed a higher number of errors per trial while showing an increase of trial duration (by almost 50%) and path length. The prolongations were particularly dependent on the inactivity phase, while their movement speed and smoothness were comparable to those of the young participants. It was suggested that this pattern might be due to a motor planning and/or a sequencing deficit of the task ([58–60]). Subjects of the frail group did not show this prolongation or increased phases of inactivity (1.0 – RA) in the current experiment, although RA appeared to be a significant predictor in the model of MLR in TEA (frailty score 0–5). Still, cognitive factors may not be the primarily limitation of performance in pre-frail or frail participants if compared to the robust participants. This seems to be in line with equal MMSE scores between the groups (*p* = 0.11, η^2^_p_ = 0.167). Additionally, we excluded participants with lower MMSE scores than 24 to avoid comprehension problems of the tasks. However, it is questionable whether recording the MMSE alone is sufficient for the assessment of the wide range of different cognitive abilities, as other cognitive functions, such as executive functions, might also play an important role (e.g., Trail Making Task (TMT) [35]). Our findings support the assertion of Panhwar and colleagues [24] that, for example, time-based assessments do not necessarily represent the characteristics of the movements and the underlying causes for changes and that, therefore, quantifying movement characteristics using kinematic analyses can be crucial for measuring the extent of frailty.

From the perspective of motor control, movement arises from a close interaction between the individual, the task, and the environment [61]. Consequently, the kinematic variables may reflect individual manifestations of frailty since the task in our experiment was standardized with stable constraints of task and environment. The between-group difference in motor performance (at natural speed) detected by accelerometer-derived measures showed a rather noticeable monotonous behavior in frail participants. This is notable, as the tasks were performed at a natural pace and did not aim to challenge the individual’s performance limits. Nevertheless, the analysis revealed clear differences between the groups without a concomitant difference in task duration. This might be comparable to previous findings of our work group where we investigated natural vs. maximal execution speed of ADL between young, old, and retirees. The results showed that the retirees were not able to decrease their trial duration or improve any other kinematic parameter in the fast condition in comparison with the natural condition. This might be based on the assumption that they were already executing the task at their maximum kinematic capacities [35]. This may also have been the case for the frail elderly in our current study. Furthermore, differences in motor performance between frailty levels are in line with literature [25, 43], however, these research groups aimed to predict the Fried score by performing tasks at a fast pace and did find differences in velocities and durations. For example, Toosizadeh and colleagues [43] introduced a quick, simple upper extremity task to categorize frailty levels. Their analyses demonstrated that the speed of elbow flexion showed the largest effect size to distinguish between robust and pre-frail older adults. Power of movement, on the other hand, had the largest effect size for the differentiation between the level of pre-frail and frail. Likewise, Kubicki and colleagues [25] involved arm motion for identifying frailty. The participants were asked to perform a rapid focal arm-raising movement, pointing to a stimulus in standing posture, while their balance was measured using a force platform. Compared to non-frail elderly, the velocity profiles of the hands were flattened. Hand movements were slowed down with longer hand movement times and lower hand peak velocities. Additionally, time peak velocities were longer for the frail group compared to the control group. However, the prefrail category was excluded. In comparison, our results showed that for TEA, PPS and for GARDEN STD, MPA and MAX95 differed between robust and frail. The parameters RA, RATIO and MPA (only for GARDEN) showed additional differences between robust und pre-frail. None of the parameters revealed differences between pre-frail and frail, though. The present study shows that without instructing maximum speed but rather emphasizing natural behavior, frail condition can be differentiated from robust and conditions with adequate kinematic measures. The parameter trial duration, however, might not be a good estimate of natural-paced upper-limb task performance with regards to frailty.

As mentioned in the sections above, frail elderly showed more monotonous behavior compared to less frail, with no differences in trial duration and MMSE scores. Compared to problems in motor control, decreased force and power as well as attempts to save energy may be the main reason behind impaired ADL performance in frail and pre-fail participants. Furthermore, this raises questions about additional influential factors contributing to the performance. Considering the verbal feedback from the participants, the gardening task might have been influenced by motivational factors and depended strongly on the thoroughness and interests of each individuum. Furthermore, participants were classified according to an adopted version of the Fried score, which mainly covers the physiological aspect of the construct of frailty. Levers and colleagues [8] stated in their review that, although many different definitions of frailty exist, physical factors, aging, and disease are the three main contributing factors in theoretical and research literature. However, there are other opinions suggesting to include physical, cognitive/psychological, socio-economic, nutritional, and social factors as well as disease and aging as a reflection of bio-psycho-social-spiritual view of health [62]. We do not know whether our measures would also differentiate levels of frailty if classified with different or additional factors. However, since ADL performance requests many other performance aspects than physical power and endurance, we speculate this would be the case. In fact, the amount of variance of the adapted Fried score that could be explained by our data was not very high (R^2^ = 25%) leaving room for many other contributing factors.

In our cohort, 46% of the participants were robust, 41% pre-frail, and 13% were classified as frail. While BMI, MMSE and gender showed no differences between the groups, there was a significant age difference between the robust (min – max: 71–90, mean: 78.4 years) or pre-frail (min – max: 68–89, mean: 79.8 years) group compared to the frail participants (min – max: 86 – 96, mean: 89.8 years). However, the repeated MANCOVA with age added as covariate revealed no significant impact of age. In addition, participants up to 90 years of age are also represented in the robust group, and the pre-frail and frail group did not differ in most measures despite the difference in age. Therefore, the age difference was probably not critical for our results. The age differences between groups is, however, in line with literature stating that this is a status closely associated with ageing and is consistent with Fried’s statement that, on average, those who were frail were older than those who were not frail or were in the intermediate group. Additionally, Mitnitski et al. [63] illustrated that the accumulation of deficits was shown to increase monotonically with chronological age, and proposed measuring the frailty index FI (different frailty measure) as a proxy of aging. Levers and colleagues [8] stated in their review, that study populations chosen suggest a belief in the existence of a relationship between frailty and aging as all participants were over the age of 60. This of course implies a relation between frailty and aging. However, the relationship between frailty and aging and how this relates to daily life performance must be clarified in further studies.

Despite many positive findings, this study includes several limitations that need to be addressed. First, in this experimental cohort study, we did not use the original Fried frailty score. Nevertheless, the five characteristics were observed and their construct and predictive validity demonstrated elsewhere [49]. Second, as stated above, the number of errors being made during the execution of a task increases with age [e.g., 30]. We did not control for errors during both ADL tasks. However, as the number of errors increases, the execution time would be expected to increase too if errors were corrected by the subjects. Third, as stated in our methods section, the absolute acceleration vector was calculated by the Euclidean mean and the gravitation was subtracted as a constant. This can lead to errors, particularly during low horizontal accelerations. Our results were, however, not substantially impacted by these errors and appeared to be valid but are still in the need for simulations. Additionally worth mentioning is the problem that known and standardly collected kinematic parameters, such as path length and velocities [22, 57], cannot be calculated precisely based on acceleration measures. In future studies, the addition of gyroscope data could be promising [64]. Fourthly, the smartwatch was attached to the dominant wrist of the participants. Hand dominance, however, was only assessed by verbal information without using standardized questionnaires like the Edinburgh Handedness Inventory (EHI) [65]. Lastly, as mentioned elsewhere [46], data were collected unilaterally in two highly complex bimanual ADL tasks. Therefore, an assessment of upper limb bimanual performance was not possible. This should have added interesting information as older adults seem to develop strategies to compensate for their reduced motor capacity, resulting in, among other things, less motor asymmetry and more equal performance of both hands ([e.g., 66]). This raises the question of how frailty might affect these bimanual interactions. However, unilateral measurement of the dominant upper limb seems to be an important first step in real-world application. In our experiment only two exemplary ADL tasks were tested. Therefore, the measured parameters should be tested in different ADL tasks and/or over a longer period of time, as frailty is a syndrome constantly present, but assumes a dynamic process which is characterized by frequent transitions between frailty states over time [6].

## Conclusion

In summary, this experimental cohort study showed that pure time-based measures, like trial duration, may not be a proper parameter to assess ADL motor performance in older adults with and without frailty. However, some of the calculated parameters (e.g., STD, PPS and MPA) seem to be good measures showing differences between frailty levels even in natural-paced ADL tasks. Therefore, kinematic parameter gathered during upper-extremity ADL tasks might have the potential to give further information on the motoric status of older adults with different stages of frailty or in other aspects of aging or in neurological diseases. Furthermore, assessments based on accelerometry avoid the need for direct observation or video recording and thereby save time. Further research should analyze the reliability of the measured parameters over a longer period (several consecutive days) as kinematics have the potential to give additional information about the temporal dynamics of an individual’s upper extremity status.

## Data Availability

All datasets generated and/or analyzed during the current study are available from the corresponding author on reasonable request.

## Abbreviations

ADL: Activities of daily living
F: Frail
P: Pre-frail
R: Robust
i-ADL: Instrumental activities of daily living
b-ADL: Basic activities of daily living
OR: Odds ratio
CI: Confidence interval
IMUs: Inertial measurement units
e.g.: For example
MMSE: Mini Mental State Examination
BMI: Body mass index
TUG: Timed up & go test
TD: Trial duration
RA: Relative activity
STD: Peak standard deviation
PPS: Peaks per second
RATIO: Peak ratio
SUM: Weighted sum of acceleration per second
APS: Acceleration per second
MPA: Mean peak acceleration
MAX95: 95^Th^ percentile of acceleration peaks
EHI: Edinburgh Handedness Inventory
MLR: Multiple linear regression
VIF: Variance inflation factor
TMT: Trail making task
FI: Frailty index
